# Exploring Intra-Articular Administration of Monoclonal Antibodies as a Novel Approach to Osteoarthritis Treatment: A Systematic Review

**DOI:** 10.3390/biomedicines12102217

**Published:** 2024-09-28

**Authors:** Amarildo Smakaj, Elena Gasbarra, Tommaso Cardelli, Chiara Salvati, Roberto Bonanni, Ida Cariati, Riccardo Iundusi, Umberto Tarantino

**Affiliations:** 1 Department of Biomedicine and Prevention, “Tor Vergata” University of Rome, Via Montpellier 1, 00133 Rome, Italy; amarildo.smakaj@gmail.com; 2 Department of Orthopaedics and Traumatology, “Policlinico Tor Vergata” Foundation, Viale Oxford 81, 00133 Rome, Italy; gasbarra@med.uniroma2.it (E.G.); tommasocardelli1993@gmail.com (T.C.); chiarasalvati95@yahoo.it (C.S.); riccardo.iundusi@uniroma2.it (R.I.); umberto.tarantino@uniroma2.it (U.T.); 3 Department of Clinical Sciences and Translational Medicine, “Tor Vergata” University of Rome, Via Montpellier 1, 00133 Rome, Italy; 4 Department of Systems Medicine, “Tor Vergata” University of Rome, Via Montpellier 1, 00133 Rome, Italy; ida.cariati@uniroma2.it; 5 Centre of Space Bio-Medicine, “Tor Vergata” University of Rome, Via Montpellier 1, 00133 Rome, Italy

**Keywords:** osteoarthritis, injections, intra-articular, biological products, monoclonal antibodies, animal models, systematic review

## Abstract

Biological drugs, including monoclonal antibodies, represent a revolutionary strategy in all fields of medicine, offering promising results even in the treatment of osteoarthritis (OA). However, their safety and efficacy have not been fully validated, highlighting the need for in-depth studies. Therefore, we provided a comprehensive systematic review of the intra-articular use of monoclonal antibodies for the treatment of OA in animal models, reflecting ongoing efforts to advance therapeutic strategies and improve patient outcomes. A systematic literature search was conducted in December 2023 following the PRISMA guidelines, using the Web of Science, Google Scholar, and PUBMED databases. Out of a total of 456, 10 articles were included in the study analyzing intra-articular antibodies and focusing on various targets, including vascular endothelial growth factor (VEGF), nerve growth factor (NGF), interleukin 4-10 (IL4-10), tumor necrosis factor α (TNF-α), a disintegrin and metalloproteinase with thrombospondin motifs 5 (ADAMTS5), and matrix metalloproteinase 13 (MMP-13). Most studies administered the antibodies weekly, ranging from 1 to 10 injections. Animal models varied, with mean follow-up periods of 8.9 ± 4.1 weeks. The methods of assessing outcomes, including pain and morpho-functional changes, varied. Some studies reported only morphological and immunohistochemical data, while others included a quantitative analysis of protein expression. In conclusion, monoclonal antibodies represent a promising avenue in the treatment of OA, offering targeted approaches to modulate disease pathways. Further research and clinical trials are needed to validate their safety and efficacy, with the potential to revolutionize the management of OA and reduce reliance on prosthetic interventions.

## 1. Introduction

Osteoarthritis (OA) is one of the most widespread causes of disability around the world. It affects an estimated 240 million individuals, including 10% of men and 18% of women from the age of 60 years [1]. Aside from the effects on physical health, data from the Ostheoartritis Initiative (OAI) study demonstrated that OA also had a massive impact on the development of depressive symptoms [2,3]. The implant of joint prosthesis is the main therapy but relates to the risk of postoperative complications and the necessity of a revision surgery. Based on the 2000–2019 total volume counts (data from the CMS Medicare Part-B National Summary), the log-linear exponential model forecasts an increase in rTHA (revision Total Hip Arthroplasty) of 42% by 2040 and 101% by 2060, as well as an estimated increase in rTKA (revision Total Knee Arthroplasty) of 149% by 2040 and 520% by 2060 [4]. While robot-assisted knee arthroplasty has demonstrated improvements in the pain control and functional recovery of osteoarthritis patients, the exploration of less invasive alternatives, such as intra-articular monoclonal antibodies [5], offers a promising approach to managing symptoms and delaying the need for surgery.

The current role of disease-modifying osteoarthritis drugs (DMOADs) is not simple to define because there is a significant effort to develop new therapeutic strategies to change the natural history of OA [6]. Studies on intra-articular therapies like hybrid hyaluronic acid injections have shown significant improvements in pain and disease severity in knee osteoarthritis, highlighting the potential of intra-articular treatments [7,8], which can be further advanced by exploring monoclonal antibodies. The use of blood derivatives (PRP) seems to be the most promising and effective regenerative therapy nowadays because it is considered as a simple, safe, and minimally invasive strategy for providing bioactive molecules that can influence the joint environment, promoting homeostasis restoration and possibly tissue regeneration [9,10]. However, the growing comprehension of the pathogenesis of OA, in particular the role of cytokines, growth factors, and signaling molecules [11], is opening up new perspectives for cartilage repair and treatment. In 2023, Rodriguez-Merchan et al. described the current role of DMOADs, highlighting their potential in managing OA and their challenges [12]. Particularly, the manuscript discussed various approaches, including anti-cytokine therapies (e.g., tanezumab, adalimumab), enzyme inhibitors, growth factors, gene therapies, peptides, and other novel treatments. It highlighted that tanezumab could reduce hip and knee pain but may lead to serious side effects like osteonecrosis and the increased need for joint replacement surgery, especially when combined with non-steroidal anti-inflammatory drugs (NSAIDs) [13]. Lorecivivint, administered via intra-articular injection, was also mentioned as safe and well tolerated without significant systemic complications [14]. Although these data are preliminary, increasing evidence suggests that biologists are revolutionizing treatment approaches in many other medical fields, significantly improving patient outcomes for conditions such as severe asthma, ulcerative colitis, and Crohn’s disease [15]. They also play a crucial role in managing severe autoimmune conditions in rheumatology and have introduced monoclonal antibodies for T-cell lymphomas [16] and checkpoint inhibitors for metastatic melanoma in oncology [17].

Based on this evidence, the goal of this comprehensive article is to offer an updated and thorough systematic review of the evidence regarding the intra-articular use of monoclonal antibodies as a potential treatment for osteoarthritis in animal models.

## 2. Materials and Methods

The systematic literature search was conducted according to the PRISMA (Preferred Reporting Items Systematic Reviews and Meta-Analysis) guidelines [18] in December 2023. The ID provided by PROSPERO is 531977. Three bibliographic databases were used for the literature search, namely Web of Science (WoS), Google Scholar, and PUBMED. The electronic research was performed using the following keywords, their MeSH terms, and the logical operators “AND” and “OR”: (“osteoarthritis” OR “arthritis*” OR “arthrosis”) AND (“antibody*” OR “monoclonal” OR “biologic* drug*”) AND (“injection*”) AND (“articular” OR “intraarticular”). The literature search was then extended by reviewing the reference lists of selected publications. The research question was based on the PICO scheme (population (P), intervention (I), comparison (C), and outcome (O)) as follows:

Do animal models of osteoarthritis (P) treated by intraarticular injection of monoclonal antibodies (I) have better outcomes, in terms of pain and function, (O) compared to standard treatment or placebo (C)?

Studies were included if they involved animal models of osteoarthritis and employed intra-articular injections of monoclonal antibodies as the primary intervention. Additionally, these studies needed to report on pertinent functional outcomes, including pain and joint function. In contrast, studies focusing on arthritis models other than osteoarthritis, such as rheumatoid arthritis, were excluded. Only research pertaining to animal models of osteoarthritis that utilized various monoclonal antibodies and encompassed diverse osteoarthritis models was selected for inclusion. Two authors independently screened titles and abstracts, as well as the collected data, from the studies meeting the inclusion criteria (A.S., T.C.). In cases of disagreement, a third author was consulted to reach a consensus (U.T). The extracted data included information on the animal model, monoclonal antibodies used for treatment, arthritis model, injection details (number, volume, concentration), functional outcomes, complications, and follow-up. Non-English papers were excluded from the review. Expert opinions, letters to the editor, unpublished studies, case reports, case series, abstracts presented at scientific conferences, in vitro research, and chapters from books were also excluded. It was not feasible to include articles with unavailable full text in the study. No date of publication limits was set. We used Microsoft Excel (Microsoft^®^ Excel^®^ for Microsoft 365 MSO—Version 2408, 64-bit) to tabulate the collected data. The categorical variables were displayed as frequencies and percentages, while continuous variables were presented as means and standard deviations. The numerical values in the report were rounded to one decimal digit.

## 3. Results

**Figure 1:**
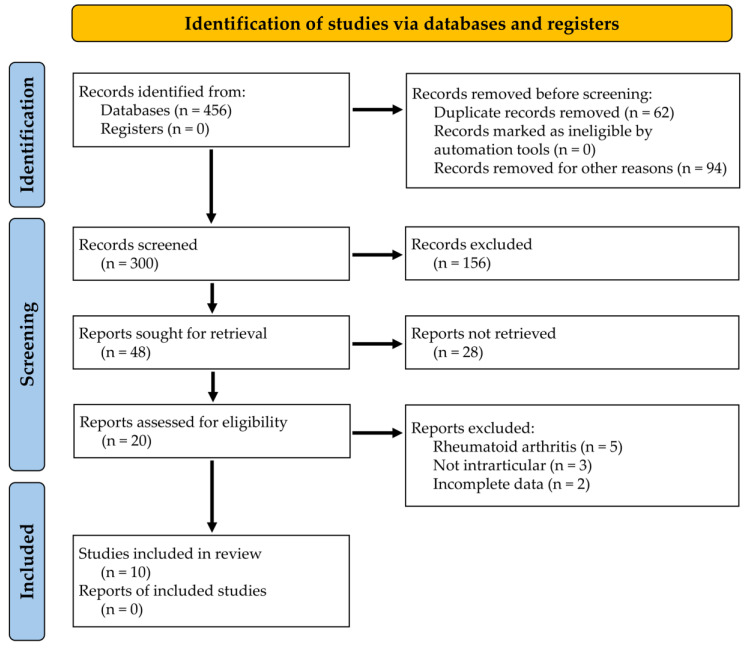
PRISMA flow chart summarizing the steps in the review process.

A total of 456 records was retrieved through an electronic search using WoS, Google Scholar, and PUBMED sources. After eliminating duplicates and non-English articles, 156 records were excluded from the study. Up to 48 articles were chosen after a review of their titles and abstracts. After thoroughly reading the complete text, a total of 10 articles were incorporated into the literature review [19,20,21,22,23,24,25,26,27,28] (Figure 1). Only research involving the use of antibodies delivered through the intra-articular pathway were considered.

The included studies used the following antibodies: three studies focused on using antibodies directed against the vascular endothelial growth factor (anti-VEGF) [20,21,22], one study evaluated the efficacy of antibodies directed against nerve growth factor (anti-NGF) [23], two studies evaluated antibodies directed against interleukin 4-10 (anti-IL4-10) [24,25], and one study investigated the role of antibodies directed against tumor necrosis factor α (anti-TNF-α) [26]. In addition, one study was conducted using antibodies directed against a disintegrin and metalloproteinase with thrombospondin motifs 5 (ADAMTS5) [28] and another used antibodies directed against matrix metalloproteinase 13 (anti-MMP-13) [19].

Most of the papers in the study involved weekly administration of intra-articular antibodies, while, in one case, there was a single drug injection [27]. The number of administrations varies from 1 to 10, with an average of 4.6 ± 3.2 and a mode of 4. Two articles did not present the volume of drug injected intra-articularly [19,23]. The mean volume of the injected drug was highly variable, with an average of 408.6 ± 371.6 µL. The concentration of antibody used varied widely between articles and even within the same paper. For example, it ranged from 0.02 mg/mL in the work of van Helvoort et al. [25] to 50 mg/mL in the study by Vadalà et al. [20], indicating that this aspect of research still requires optimization.

Four research studies employed rabbits as a study model [20,21,22,26], while rats were utilized in three instances [23,24,27], mice in two instances [19,28], and canine models in one case [25]. The number of animals used ranged from 6 to 61 (26.4 ± 16.2). The models of arthrosis used in the studies varied. Bedingfield et al. utilized compressive mechanical loading to simulate post-traumatic OA [19]. Two groups employed anterior cruciate ligament transection (ACLT) [20,22], while Zhang et al. combined the Hulth technique (medial meniscus resections) with this approach [26]. Li et al. opted for prolonged immobilization, specifically 5 weeks of plaster application [21]. The Groove model was utilized by van Helvoort in both 2019 and 2021 [24,25]. Additionally, two authors administered arthrosizing intra-articular injections [23,27], while only one author tested aged mice that exhibited signs of arthrosis [28].

The mean follow-up period was 8.9 ± 4.1 weeks. In two studies, animals were sacrificed at 24 h after the last administration [24,25], totaling a follow-up of 10 weeks from the first administration. Urech et al. is the only study where animal models had an extremely short follow-up [27]; they were sacrificed just 24 h after drug administration. No side effects were reported.

All works considered a control group, represented by the contralateral limb where sterile saline solution (SSS) was injected. No side effects were reported.

The outcomes were assessed in a heterogeneous manner. Four papers evaluated the clinical outcomes, focusing on pain [22,23,24,25]. The assessment methods for pain also varied and included arm weight loss, force plate analysis, and the von Frey filament.

Morpho-functional changes were assessed in numerous ways, including immunohistochemical expression evaluation, morphological analysis with OARSI score, Western blotting, ELISA, and RT-PCR. There was no single score used to assess the severity of cartilage damage. The Mankin score was utilized in three of the selected articles [23,26,28]. Similarly, the macroscopic assessment of cartilage damage was not evaluated in all the papers included in the study; however, the OARSI score was used in three cases [20,22,28].

Four of the included papers only reported morphological and immunohistochemical data without providing semiquantitative protein expression data such as Western blotting and RT-PCR results [20,21,26,29]. Additionally, the antibodies used in immunohistochemical were not consistently the same, although MMPs were often included.

Table 1 summarizes the main information about the studies included in the systematic review.

## 4. Discussion

This comprehensive systematic review aimed to explore the potential of intra-articular monoclonal antibodies in treating osteoarthritis, a condition currently lacking effective disease-modifying interventions. The analysis covered 10 studies that looked at various antibodies including anti-VEGF, anti-NGF, anti-IL4-10, anti-TNF-α, anti-ADAMTS5, and anti-MMP-13 [19,20,21,22,23,24,25,26,27,28]. These studies represent an important initial step in understanding the therapeutic potential and optimization of monoclonal antibodies for OA treatment. However, there is limited evidence from single antibody studies which makes it difficult to confidently establish their replicability. Only the monoclonal antibodies targeting VEGF, IL4-10, and TNF-a have been studied by more than one study included in this review.

The variance in administration frequency, dosage, and concentration of antibodies underscores the nascent stage of this therapeutic approach. The average administration of 4.6 ± 3.2 times and the wide range of antibody concentrations, from 0.02 mg/mL to 50 mg/mL, highlight the need for standardized protocols to better assess efficacy and optimize treatment regimens. Moreover, the wide range of volume injected points to a lack of consensus on the optimal volume for therapeutic efficacy, reflecting the early stage of research and development in this area. In a translational context, it is important to consider that most intra-articular injections in humans have a volume of 2 mL to prevent the excessive distension of the joint capsule [30]. When determining drug concentration, it should be based on the pilot studies aiming to achieve the optimal dose/response ratio while minimizing potential side effects.

The variability in animal models, including rabbits, rats, mice, and canines, and the methods used to induce arthrosis, from mechanical loading to surgical techniques, reflects the complexity of simulating human OA and assessing treatment outcomes across different models. Hence, one drawback of the chosen research papers is that while the arthrosis models they utilize have been validated by several studies, they primarily represent a post-traumatic form of arthrosis caused by factors such as mechanical overload and medial meniscus or ACL injury. This may not accurately reflect the most common form of arthrosis known as primary arthrosis.

The absence of documented adverse reactions is encouraging, implying a possibly positive safety record for delivering antibodies intra-articularly. However, the lack of reported side effects should be interpreted carefully, as it could indicate underreporting or the preliminary nature of these studies rather than a definitive confirmation of safety.

The studies showed considerable variation in outcome assessments, including clinical factors such as pain and changes in morpho-function. The differences in assessment methods and the absence of a standardized scoring system for evaluating the severity of cartilage damage highlight the importance of establishing consensus on how therapeutic outcomes are evaluated in research on osteoarthritis.

The inclusion of control groups, where the opposite limb receives injections of SSS, enhances the credibility of the results. However, variations in the data presentation, such as the lack of semi-quantitative protein expression data in four studies, restricts our ability to make firm conclusions about the effectiveness of intra-articular monoclonal antibodies for treating OA.

One of the most promising antibodies targets VEGF. Studies have shown increased vascularization in both the synovial membrane and subchondral bone during AO, suggesting a potential role of angiogenesis in its pathogenesis [31,32].

It has been found that anti-VEGF monoclonal antibodies, such as bevacizumab, showed potential in arresting the progression of osteoarthritis in animal models [30]. Histological evaluation showed that bevacizumab treatment reduced Safranin O loss, fissures, erosions, and chondrocyte loss in OA animals [20]. In the study by Vadalà et al., the treatment groups had good staining retention, smooth articular surfaces, and no notable changes in cell density or disposition. Quantitative assessment confirmed a significant difference in cartilage degeneration between the untreated OA group and all treatment groups using the OARSI scoring system [20]. Histological analysis of synovial tissues also revealed a reduction in inflammation with bevacizumab treatment compared to untreated controls. Additionally, intra-articular bevacizumab upregulated type 2 collagen and aggrecan expression while downregulating MMP-13 expression in the articular cartilage compared to the untreated controls [20].

Moreover, Nagai et al., in 2014, also reported similar findings regarding the anticatabolic properties of bevacizumab, supporting the potential of monoclonal antibodies as a treatment for osteoarthritis [22]. Histological examination showed that bevacizumab did not have adverse effects on normal joints and led to an increase in the expression of collagen type 2 in the articular cartilage, reducing degeneration. Real-time PCR analysis supported these findings by indicating lower expression of catabolic factors in the synovium of the intravenous group compared to the OA group. Additionally, both the intra-articular and intravenous administration of bevacizumab exhibited beneficial effects, but histological evaluation and pain assessments favored IA administration 12 weeks post-administration despite receiving a smaller dosage than IV [22].

In 2019, Li et al. also explored the use of bevacizumab. The study demonstrated the positive effects of the anti-VEGF monoclonal antibody on reducing cartilage degeneration and inflammation and increasing the expression of key cartilage components, such as aggrecan and collagen type 2 [21]. Furthermore, the localized administration of monoclonal antibodies, such as intra-articular administration of bevacizumab, appears to be a more advantageous approach compared to systemic administration, considering the dosage and potential adverse effects [21]. Moreover, some results from an ex vivo cartilage explant culture showed that bevacizumab reduced the levels of cartilage degradation markers, particularly in the presence of IL-1β. Additionally, bevacizumab increased the expression of collagen-related genes, indicating a protective and anabolic effect on cartilage. These findings suggest that bevacizumab may help slow OA progression [33].

Using antibodies targeted at NGF also appears to be a promising approach for treating symptoms, as pain is the most disabling manifestation in OA patients [34,35,36]. In fact, tanezumab, a humanized monoclonal antibody targeting NGF, showed potential in managing pain and improving function in patients with knee osteoarthritis [13,37]. A meta-analysis including ten relevant studies demonstrated that tanezumab was more effective than placebo in reducing pain, improving physical function, and enhancing patient’s global assessment. Although the adverse events were higher in the tanezumab groups, they were generally well tolerated [38]. These findings contribute to evidence supporting nerve growth factor targeting for osteoarthritis pain management while highlighting the need for vigilance regarding side effects [13].

From the present review, it has emerged that anti-NGF injection effectively alleviated pain in OA model rats, as shown by the improved weight-bearing performance from week 3 onwards in the study by Tian et al. However, anti-NGF did not improve allodynia induced by MIA injection at any concentration tested. Additionally, NGF antibody injection showed no adverse effects on the joints or cartilage pathology based on histological evaluations. However, a systematic review on the efficacy of TNF inhibitors in hand osteoarthritis, administered subcutaneously rather than intra-articularly, found no significant impact on pain or grip strength over the short-term (4–6 weeks) or longer-term (12 months) periods. However, some evidence suggests that TNF inhibitors may slow the progression of structural damage in patients with inflammatory signs, though the overall certainty of the evidence remains low [39].

In a similar way, the role of interleukin-1 (IL-1) as a therapeutic target in osteoarthritis (OA) has been under investigation for many years due to its involvement in cartilage degradation and synovitis. However, recent large randomized controlled trials have shown limited success, leading to a decline in enthusiasm for IL-1 as a viable target in OA treatment [40].

Upon further exploration of the potential of monoclonal antibodies in osteoarthritis treatment, it has become evident that the mechanisms of action of these antibodies extend beyond mere symptom management. The ability of monoclonal antibodies to specifically target key pathways involved in osteoarthritis pathogenesis represents a significant advancement in many fields and provides a potential breakthrough in the treatment of osteoarthritis.

Monoclonal antibodies are already widely used locally in various areas, including topical application in dermatology [17] and intraocular use in ophthalmology [41]. Monoclonal antibodies already play a significant role in ophthalmology due to their clinical effectiveness and the advancing understanding of their pharmacological properties. Biologics targeting vascular endothelial growth factor have transformed the treatment of age-related macular degeneration (AMD), a major cause of blindness in older adults [41]. The literature still contains very few clinical trials that have yielded satisfactory results with the use of monoclonal antibodies [36]. In contrast, numerous studies have focused on the systemic use of these drugs [42,43,44]. Additionally, the intra-articular use of monoclonal antibodies has been investigated for the treatment of rheumatoid arthritis [45,46], but this is outside the scope of this work.

The study has various limitations, including the diversity of animal models and techniques used to induce arthrosis. In addition, there is considerable variation in the timing, quantity, and strength of treatment, as well as in the assessment methods used to evaluate outcomes across different studies. Moreover, an assessment of the risk of bias or distortion was not conducted in this review, and this should be considered when interpreting the findings and conclusions. However, this study offers several advantages, including consideration of numerous antibodies and the demonstration of the safety profile of these drugs for intra-articular use with evidence of the absence of serious side effects.

The article’s translational potential lies in the myriad of questions it raises from a translational perspective. These encompass not only intricately biological aspects, such as the efficacy of monoclonal antibodies for intra-articular use (including the preferred type of antibody and proposed stage of arthrosis for treatment) but also technical considerations like concentration, volume, number of administrations, and frequency of the treatment. Additionally, the work indicates a likely high safety profile for these drugs due to the absence of stated adverse effects. Overall, there is significant translational potential in utilizing monoclonal drugs for arthrosis therapy. This could potentially reduce the need for surgery among patients and provide an alternative therapeutic option to prostheses for those deemed inoperable due to systemic comorbidities.

## 5. Conclusions

The safety profile is extremely favorable, with encouraging findings in preclinical studies. It is essential to commence phase I clinical trials to examine the safety of monoclonal antibodies when used intra-articularly for osteoarthritis. Assessing individual antibodies at standard concentrations and volumes will provide crucial insights into their safety in human subjects. Additionally, it is important to investigate the potential conjugation of these antibodies with the commercially available hyaluronic acid solutions. This approach holds promise for enhanced therapeutic efficacy and prolonged drug release within the joint space.

## Figures and Tables

**Table 1:** Overview of the essential information from the studies included in the review.

First Author	Year	Animal Model	Number of Animals	Control Group	OA Model	Type of Antibody	Concentration	Volume	Frequency	Number of Injections	Follow-Up	Outcomes
Bedingfield et al. [19]	2021	mice	9	Y	CML *	Anti-MMP-13	0.5 mg/mL	-	weekly	6	6 w	MMP13 ↓
Vadalà et al. [20]	2021	rabbit	18	Y	ACL ^§^	anti-VEGF	6.25 to 25 mg/mL	800 μL	weekly	4	12 w	MMP13 ↓, coll II ↑, aggrecan ↑, OARSI ↑
Li et al. [21]	2019	rabbit	8	Y	5 w plaster	anti-VEGF	10 mg/mL	400 μL	3 weeks	2	6 w	MMP1 ↓, VEGF ↓
Nagai et al. [22]	2014	rabbit	6	Y	ACLT ^§^	anti-VEGF	25 mg/mL	1000 μL	weekly	4	12 w	Pain ↓
Tian et al. [23]	2021	rat	18	Y	MIA ^†^ injection for 2 weeks	anti-NGF	-	-	weekly	4	-	Pain ↓
van Helvoort et al. [24]	2021	rat	10	Y	Groove model	anti-IL4-10	0.02 mg/mL	25 µL	weekly	10	10 w	Pain ↓
van Helvoort et al. [25]	2019	canine	4	Y	Groove model	anti-IL4-10	0.02 mg/mL	500 μL	weekly	10	10 w	Pain ↓
Zhang et al. [26]	2012	rabbit	20	Y	Hulth technique (medial meniscus resections) ACLT	anti-TNF-α	10 to 20 mg/mL	500 μL	weekly	3	12 w	Mankin ↑
Urech et al. [27]	2010	rat	6	Y	TNF-α injection	anti-TNF-α	-	40 μL	-	1	48 h	-
Chiusaroli et al. [28]	2013	mice	41	Y	Old mouse	anti-ADAMTS5	0.3 to 3 mg/mL	4 μL	6 weeks	2		Mankin ↑, OARSI ↑

* Compressive Mechanical Loading, ^§^ Anterior Cruciate Ligament Transection, ^†^ Monoiodoacetate, ↑ Increase, ↓ Reduction.

## Data Availability

Template data collection forms, data extracted from included studies, and analytic code are available under reasonable request to the Authors.
